# Editorial: Women in microbial physiology and metabolism: 2023

**DOI:** 10.3389/fmicb.2024.1437794

**Published:** 2024-06-14

**Authors:** Maria Filippa Addis, Jie Xiao, Ilana Kolodkin-Gal

**Affiliations:** ^1^Department of Veterinary Medicine and Animal Science, University of Milan, Milan, Italy; ^2^Department of Biophysics and Biophysical Chemistry, Johns Hopkins School of Medicine, Baltimore, MD, United States; ^3^Scojen Institue for Synthetic Biology, Reichman University, Herzliya, Israel

**Keywords:** metabolism, microbial diversity, microbial genetics, microbial physiology, antibiotics, food microbes, women scientists

There is overwhelming evidence that female scientists have shaped modern microbiology in general and the study of microbial physiology and metabolism. In 1928, Marjory Stephenson isolated a bacterial enzyme, lactic dehydrogenase, from *Escherichia coli*. In the 1930s, the German-Jewish bacteriologist Emmy Klieneberger-Nobel developed a unique nutrient agar blend and culturing technique that allowed the bacteria that caused bronchopneumonia in rats and mice to grow in a lab for the first time and later isolated and identified several pathogenic species of mycoplasma—*Mycoplasma arthritidis* and *Mycoplasma pneumoniae*. In 1944, Elizabeth Bugie, an American microbiologist and biochemist, contributed significantly to the breakthrough discovery of the antibiotic streptomycin. Besides these fundamental discoveries, female scientists have also contributed overwhelmingly to the evolution of molecular approaches, the fundamental understanding of bacterial genetics, and the development of accurate tools to study the physiology of microorganisms. These scientists span from the unsung hero of her time, Rosalind Franklin, who played a vital role in generating an X-ray diffraction analysis of the DNA's double-helical structure, leading to the resolution of the double helix, to the American Jewish scientist Esther Lederberg, who discovered the Lambda (λ) phage and gene transfers between bacteria through a process called “specialized transduction,” and to recent microbiologists Jennifer Doudna and Emmanuelle Charpentier who won the Nobel prize for CRISPR, a bacterial immune system for self-defense, in 2020 (Nature Microbiology, 2022). Out of unending respect for the contributions of female scientists to the field, we had the distinct honor of editing this year's Research Topic of Women in Metabolism and Physiology. Accepted manuscripts in this Research Topic represent a collection of outstanding works led by female scientists in the field, including but not limited to the investigation of microbial strategies for resistance, communication, cooperation, and survival in extreme environments, the application of novel physical chemistry and omic approaches to resolving microbial metabolism, and a novel discovery that *B. subtilis* histone-like protein is involved in antibiotic tolerance. Below, we provide a glimpse of these exciting studies.

Xenobiotics often challenge the principle of microbial infallibility. One example is acesulfame. Introduced in the 1980s as a zero-calorie sweetener, it was recalcitrant in wastewater treatment plants until the early 2010s. By studying acesulfame metabolism in alphaproteobacterial degraders of the genera *Bosea* and *Chelatococcus*, Bonatelli et al. experimentally confirmed the previously postulated route of two subsequent hydrolysis steps of acesulfame to acetoacetate and sulfamate *via* acetoacetamide-N-sulfonate. By applying comparative genomics, heterologous expression, and biochemical analysis, the authors identified genes responsible for acesulfame anion degradation and surveyed their distributions in public sequence databases. The authors discuss that this pathway may be established by combining preexisting catabolic genes; horizontal gene transfer may facilitate its fast distribution. The authors conclude that structurally related natural and anthropogenic compounds, such as aminoacyl sulfamate ribonucleotide or sulfonamide antibiotics, may have supported the evolution of the acesulfame degradation pathway.

The study by González-Orozco et al. explored microbial metabolism in the context of bacterial interactions. The research delves into the interactions between the probiotic lactic acid bacterium (LAB) *Lactobacillus kefiranofaciens* from Kefir and the yeast *Kluyveromyces marxianus*. The findings suggest that co-culturing these two microorganisms enhances their survival in gastrointestinal conditions and improves their adhesion to epithelial cells. These findings indicate that the combination enhances the probiotic potential of both species. The authors highlight the potential of using co-cultures of yeast and lactic acid bacteria in creating novel fermented functional products that support human health. The contribution of the food microbiome to our health is also manifested in a review by Eicher et al. where the role of citrate is thoroughly discussed with the LAB bacterium *Oenococcus oeni*, which is involved in the second step in wine fermentation.

Haenelt et al. provide an interesting insight into the communal genetics of wastewater. In their research, the authors shed light on antibiotic-resistant bacteria, their resistance genes (ARGs), and their dynamics in untreated and treated wastewater. The authors reveal that higher proportions of wastewater rivers increased the absolute and relative abundance of ARGs encoding for sulfonamide resistance genes and that this effect was season-dependent. Moreover, they obtain evidence that biofilms in wastewater may serve as a reservoir for ARGs, in agreement with the dense growth of microorganisms in a biofilm, where limited diffusion of small molecules may provide biofilm microenvironments with altered antibiotic concentrations. The community contribution to genomic evolution toward environmental resistance is also reflected in Pagnucco et al., who explore metal tolerance and biosorption of four bacterial environmental strains of *Serratia, Raoultella*, and *Klebsiella* isolated from Saint Clair River sediments. The effective removal of various metal cations (As^3+^, Pb^2+^, Cu^2+^, Mn^2+^, Zn^2+^, Cd^2+^, Cr^6+^, and Ni^2+^) by these strains confirmed that that metal absorption involves interactions between metal ions and functional groups on the surface of the strains. Moreover, the study revealed a variation in specifications of metal absorption between strains, highlighting the importance of tailoring selected strains and species to suit the environment's metal composition.

Our understanding of microbial metabolism fundamentally relies on technologies that can sensitively and accurately measure metabolites. Kassem et al. review how Fourier Transform-Infrared (FT-IR) spectroscopy can be a powerful tool to resolve the physiology of microorganisms and their responses to the environment. The applications of FT-IR spectroscopy open new fronts in analyzing microbial composition, functions, and interactions and have significantly advanced our understanding of microbial communities and their interactions within complex ecosystems. Key challenges in harnessing the full potential of FT-IR are thoroughly discussed. The review describes how qualitative insights into microbial composition and functional groups can be obtained with FT-IR spectroscopy and provides a comprehensive summary of data interpretation, analysis, and handling. Moreover, the authors discuss future aspects of standardization of FT-IR spectroscopy for microbial identification and the integration of FT-IR with complementary techniques, such as Raman spectroscopy, mass spectrometry, and genomics, which may revolutionize the study and resolution of complex metabolic pathways and molecular interactions.

Bacterial communication is a fundamental process synchronizing gene expression and collective behavior within the bacterial population. The most studied bacterial communication system is quorum sensing, a cell density system in which the concentration of inducers increases to a threshold level, allowing detection by specific receptors. As a result, bacteria can change their behavior in a coordinated way. In *Pseudomonas* spp., quorum sensing based on the synthesis of N-acyl homoserine lactone molecules is well studied. However, volatile organic compounds, although considered communication signals in the rhizosphere, are understudied. In their research article, Dupont et al. explore the role of the volatile organic compound 1-undecene in the communication of *Pseudomonas fluorescens* MFE01, a strain with a very active Type VI secretion system that can kill some competitive bacteria, by constructing defective mutants. Using this approach, they demonstrate that *P. fluorescens* MFE01 uses 1-undecene emission for aerial communication, reporting this volatile organic compound as a bacterial intraspecific communication signal for the first time.

*Pectobacterium* is considered one of the top ten bacterial pathogens that causes harvest loss of potatoes and other vegetables in the field and during transport or storage. These bacteria can cause soft rot disease in various plants due to the secretion of cell wall degrading enzymes. *Pectobacterium betavasculorum* is a member of the *Pectobacerium* genus that inhabits a variety of niches and is found in all climates. It is responsible for the vascular necrosis of sugar beet and soft rot of many vegetables. In their original research article, Smoktunowicz et al. investigated the metabolism of *P. betavasculorum* in the presence of sucrose and xylose, the two main sugars of sugar beet and artichoke, by applying untargeted metabolomics coupled with genomics. The authors confirmed the ability of the strains to use various sugars as the only carbon source, and provided information on the active metabolic pathways for their degradation.

Extreme environments are challenging for life, yet bacteria have developed strategies for survival. The Atacama Desert, located in northern Chile at the border with Bolivia and Argentina, is the driest, with the highest radiation, and one of the most ancient deserts in the world. In their original research article, Reverdy et al. provide a comprehensive picture of microbial diversity and investigate the survival strategies of bacteria living in this hostile habitat. Upon sampling 18 locations and cultivating 74 unique isolates, the authors characterized pigment production, biofilm formation, production of inhibitory substances, and antibiotic resistance as probable survival mechanisms, providing insights into bacterial diversity and the strategies that bacteria use to survive in extreme environments. These functional characterizations create opportunities for their exploitation in agriculture, healthcare, or biotechnological applications.

Lastly, Carr et al. reveal a novel role of a histone-like protein in microbial tolerance to antibiotics. Unlike archaea and eukaryotes, in which DNA is wrapped around in a nucleosome-like structure, bacteria use histone-like proteins to control the condensation of the microbial chromosome. In *Bacillus subtilis*, the histone-like protein HBsu is acetylated at seven sites, which regulates DNA compaction and the process of sporulation. The authors analyzed a collection of HBsu mutants to reveal the potential roles of this protein in response to antibiotics. The authors demonstrated that the acetylation status of HBsu led to an increase in persister cell formation. Moreover, deacetylation-mimic mutants with compacted nucleoids delayed in resuming growth after antibiotic removal. This work suggests that histone acetylation is required to escape the persistent state, which may play an important role in the epigenetic machinery of bacteria in the response to antibiotics.

Collectively, these discoveries highlight the significant contributions of femal scientsits to the field of microbial sciences with their broad scope and depth, emphasizing the importance of diversity and inclusion in our scientific community.

## Author contributions

MA: Writing – original draft, Writing – review & editing. JX: Writing – original draft, Writing – review & editing. IK-G: Writing – original draft, Writing – review & editing. All authors contributed equally to this work.
